# Gelatin-based nanoparticles and antibiotics: a new therapeutic approach for osteomyelitis?

**DOI:** 10.3389/fmolb.2024.1412325

**Published:** 2024-07-30

**Authors:** Ali Sherafati Chaleshtori, Zeynab Marzhoseyni, Negin Saeedi, Rosita Azar Bahadori, Samaneh Mollazadeh, Hossein Pourghadamyari, Esmaeil Sajadimoghadam, Kazem Abbaszadeh‐Goudarzi, Amin Moradi Hasan-Abad, Reza Sharafati Chaleshtori

**Affiliations:** ^1^ Medical Plants Research Center, Basic Health Sciences Institute, Shahrekord University of Medical Sciences, Shahrekord, Iran; ^2^ Department of Orthopedics, Faculty of Medicine, Shahrekord University of Medical Sciences, Shahrekord, Iran; ^3^ Department of Microbiology and Immunology, Faculty of Medicine, Kashan University of Medical Sciences, Kashan, Iran; ^4^ Department of Microbiology, Faculty of Biological Sciences, Islamic Azad University, North Tehran Branch, Tehran, Iran; ^5^ Department of Molecular Genetics, Parand Branch, Islamic Azad University, Tehran, Iran; ^6^ Natural Products and Medicinal Plants Research Center, North Khorasan University of Medical Sciences, Bojnurd, Iran; ^7^ Applied Cellular and Molecular Research Center, Kerman University of Medical Sciences, Kerman, Iran; ^8^ Department of Nursing, School of Nursing and Midwifery, Bam University of Medical Sciences, Bam, Iran; ^9^ Cellular and Molecular Research Center, Sabzevar University of Medical Science, Sabzevar, Iran; ^10^ Autoimmune Diseases Research Center, Shahid Beheshti Hospital, Kashan University of Medical Sciences, Kashan, Iran; ^11^ Research Center for Biochemistry and Nutrition in Metabolic Diseases, Kashan University of Medical Sciences, Kashan, Iran

**Keywords:** gelatin-based nanoparticles, osteomyelitis, antibiotics, sustained release, biocompatibility

## Abstract

The result of infection of bone with microorganisms is osteomyelitis and septic arthritis. Methicillin-resistant *Staphylococcus aureus* (MRSA) is responsible for most of its cases (more than 50%). Since MRSA is resistant to many treatments, it is accompanied by high costs and numerous complications, necessitating more effective new treatments. Recently, development of gelatin nanoparticles have attracted the attention of scientists of biomedicine to itself, and have been utilized as a delivery vehicle for antibiotics because of their biocompatibility, biodegradability, and cost-effectiveness. Promising results have been reported with gelatin modification and combinations with chemical agents. Although these findings have been suggested that gelatin has the potential to be a suitable option for continuous release of antibiotics in osteomyelitis and septic arthritis treatment, they still have not become routine in clinical practices. The most deliver antibiotic using gelatin-derived composites is vancomycin which is showed the good efficacy. To date, a number of pre-clinical studies evaluated the utility of gelatin-based composites in the management of osteomyelitis. Gelatin-based composites were found to have satisfactory performance in the control of infection, as well as the promotion of bone defect repair in chronic osteomyelitis models. This review summarized the available evidence which provides a new insight into gelatin-derived composites with controlled release of antibiotics.

## 1 Introduction

Chronic osteomyelitis treatment is considered to be challenging, since elimination of the main responsible pathogen has remained a problem for orthopedic surgeons (Kavanagh et al., 2018). Various bacteria could cause chronic osteomyelitis such as *Staphylococcus aureus*, *staphylococci*, Propionibacterium species, Enterobacteriaceae species, *Pseudomonas aeruginosa*, Salmonella species, and *Streptococcus pneumoniae* (Hatzenbuehler and Pulling, 2011). Chronic osteomyelitis is accompanied by high rates of morbidity and mortality (particularly in older patients), and disrupts the quality of life of those affected with this disease. Similar to other infections, antibiotic therapy is generally employed to manage osteomyelitis (Schwarz, 2020).

Osteomyelitis represents a significant bone infection, presenting in either acute or chronic forms. This condition entails an inflammatory reaction affecting the bone and its associated structures, triggered by pyogenic microorganisms disseminated via the bloodstream, fractures, or surgical interventions. Chronic osteomyelitis is recognized with the development of low grade inflammation, presence of bacteria and/or other microorganisms in the affected area along with pus, sequestrate, and even fistula (Lew and Waldvogel, 1997).

Mostly, osteomyelitis cause is a microorganism which arrived to the bone from adjacent infected tissue, blood or even direct inoculation due to trauma. Usually, hematogenous infections are resulted from a single microorganism and other types (direct inoculation and adjacent tissue) resulted in polymicrobial infection. The challenge in the treatment of osteomyelitis is the ability of microorganisms to live in the necrotic tissues of the bone for a substantial period of time especially if the surgical debridement has not been occurred properly (Lima et al., 2014).

Debridement of necrotic tissues and using antimicrobial agents are the common methods for treatment of osteomyelitis. Choosing the best antibiotic against the osteomyelitis-causing microorganism should be based on primary evaluations including staging, culture, and determination of susceptibility. Early initiation of antibiotics have led to the more favorable results (Cierny III, 2011; Hatzenbuehler and Pulling, 2011; Walter et al., 2012; Lima et al., 2014).

Initiation of osteomyelitis is by establishment of bacteria via different routes such as direct inoculation, hematogenous seeding or from airborne infection. By reaching to the bone, bacteria produce biofilm to protect themselves against antimicrobial agents and immune system activity such as phagocytosis. Moreover, the metabolic activity of bacteria is reduced and they change from motile forms to sessile ones. These changes increase the resistance of bacteria against different antimicrobial agents as the effective dose for killing bacteria in biofilms are about 10–100 times the standard dose which make the antibiotic therapy to the dangerous and ineffective procedure (Gogia et al., 2009). The antibiotic might not be delivered well enough, at the required concentration to remove all bacteria. Persisted cells or biofilm will therefore remain and despite surgical debridement, therapies fail in ∼20% of subjects.

**FIGURE 1:**
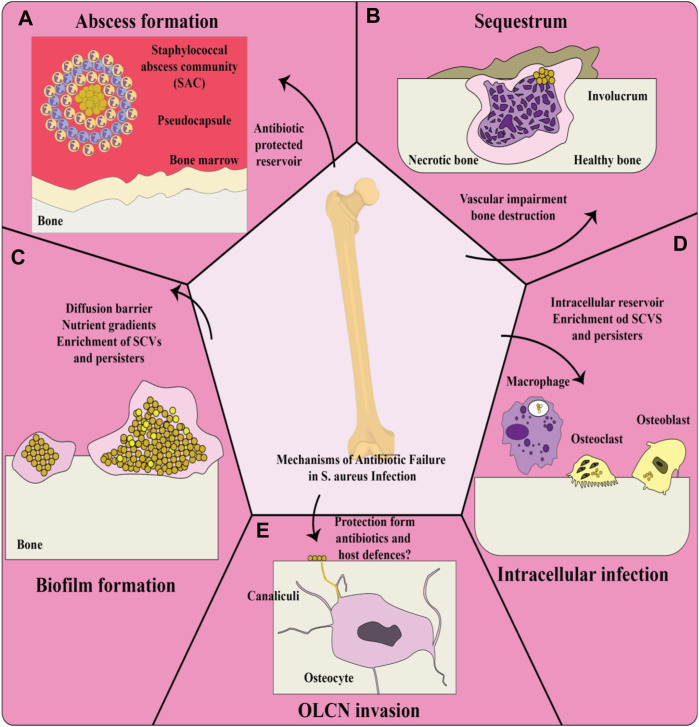
Treatment failure of *Staphylococcus aureus* osteomyelitis through multiple mechanisms; **(A)** Invasive staphylococcal infections are characterized by abscesses. Staphylococcal abscess community (SAC) is composed of bacteria inside the abscess core enclosed by a pseudocapsule containing fibrin and other extracellular matrix proteins of host, which subsequently lead to the recruitment of immune cells such as viable and non-viable neutrophils. Strong antibiotic resistance can be observed for bacteria inside a SAC. **(B)** Invading bacteria form an abscess and severe inflammation in osteomyelitis, which endangers the blood supply to the bone and therefore develops necrosis. This necrosis results in sequestra tissue lesions in chronic osteomyelitis, as a nidus during continuous infection. New bone is formed in reaction to the sequestrum, thereby forming the pathological lesion of the involucrum. The efficacy of systemic antibiotic therapy is greatly reduced due to infection-caused vascular disorders. **(C)** Bacteria achieve significantly longer persistence as a result of biofilm formation on bone within bone infection and show greater tolerance to antibiotics. The formed biofilm can prevent the diffusion and subsequent penetration of antibiotics into deeper layers. The environment of biofilm, which contains large amounts of nutrients and oxygen, intensifies the development of antibiotic-resistant bacteria, such as small colony variants [SCVs]: “pink cocci,” and persisters: “organ cocci.” **(D)** *S. aureus* invading resident bone cells (such as osteoblasts and osteoclasts) and professional phagocytes (such as macrophages) can survive inside these cells, leading to increased antibiotic tolerance because the majority of antibiotics have an extracellular action, so that the evidence showed that the intracellular host environment promotes the construction of persisters and SCVs. **(E)** Osteocytes are the main cells in the bone matrix found in lacunae structures and connected to each other through canaliculi, a 3D network of canals. Chronicity of osteomyelitis caused by *S. aureus* occurs through colonization of the osteocyte lacuno-canalicular network (OLCN) due to the absence of the antibiotic concentration required to eradicate the bacteria. Bacteria inside the OLCN may also remain hidden from the host response (Gimza and Cassat, 2021).

The complications of osteomyelitis treatment can be attributed to various reasons, some of which are i) antimicrobial resistance is widely observed, ii) biofilm production or metabolic alterations can lead to antibiotic tolerance, iii) antibiotics are unable to penetrate damaged and infected bone, and iv) antibiotic-protected reservoirs colonize in the bony substructure. For example, Figure 1 shows the failure of treatment of osteomyelitis due to *S. aureus* through multiple mechanisms outside of previously known antibiotic resistance (Gimza and Cassat, 2021). Several studies have reported multidrug-resistant strains of *S. aureus* in various types of samples (Sharafati-chaleshtori and Karimi, 2010; Silago et al., 2020; Zelmer et al., 2022).

Gelatin is a natural, biocompatible, and biodegradable biopolymer, which can perform multiple functions. It has been broadly used in the food, pharmaceutical, cosmetic, and medical industries, thanks to its beneficial mechanical and technological properties (Su and Wang, 2015). In the medical and pharmaceutical areas, gelatin has been employed for both implants and as a matrix for device coatings. However, there some disadvantages associated with the use of gelatin, such as weak mechanical properties, thermal instability, and having a slow rate of degradation. When gelatin is used in studies involving longer periods in a biological environment, such as controlled drug release, cellular adhesion and division, or wound regeneration, it is advisable that gelatin-based materials do not remain intact over the long term (Gorgieva and Kokol, 2011). In comparison to collagen, gelatin, in its hydrolyzed form, is more prone to degradation by various protease enzymes, resulting in a faster breakdown. Gelatin is formed as a denatured product of collagen under specific conditions, constituting an uneven protein mixture. Some drawbacks of gelatin could be simply avoided by gelatin modification, and the production of gelatin composites to improve the mechanical stability, biocompatibility, and bioactivity. The drawbacks of gelatin used in biomedical approaches have become less important owing to progress in manufacturing technology and our understanding of material chemistry (Bello et al., 2020). In the following sections, we discuss a variety of biomaterials based on gelatin and their use in biomedical applications such as scaffolds, drug delivery, and bone substitutes for the healing and regeneration of bones.

## 2 Gelatin for drug delivery

Extensive investigations have been carried out into the use of gelatin as a drug delivery carrier for various drug types, based on its properties as an organic biomaterial and its track record of safety in a number of medical and pharmaceutical applications. Antibacterial agents, antineoplastic cytotoxic drugs, anti-inflammatory drugs, and most recently nucleic acids and hydrophobic materials have all been reported in the literature to be advantageously delivered by gelatin-based materials (Kumar P. S. et al., 2011; Lee et al., 2013; Santoro, Tatara and Mikos, 2014). Adjusting the properties of gelatin can result in optimized drug-loading efficiency. Remarkably, the isoelectric point for gelatin can be aligned with the electrostatic properties of the drug molecule, which allows it to be employed in either alkaline or acidic conditions (Tabata and Ikada, 1998).

## 3 Preparation of gelatin nanoparticles

In the literature, gelatin nanoparticles (GNPs) have recently been described as a carrier system for drug delivery as well as gene delivery (Zwiorek et al., 2004; Changez et al., 2005; Jiang et al., 2012; Elzoghby, 2013; Zhou J. et al., 2018; Zhou X. et al, 2018). Since the first description in 1978 (Marty & RC, 1978), various methods for preparing GNPs have been described, including the techniques described below. Some advantages and disadvantages of several different preparation techniques for gelatin nanoparticles are shown in Table 1.

**TABLE 1:** Some advantages and disadvantages of several different preparation techniques for GNPs (Khan, 2020).

Preparation method	Size (nm)	Positive aspects	Negative aspects
Desolvation	200–500	Simple procedure	Agglomeration, polydispersity and stability issues
Two step desolvation	100–300	Homogeneous size	Narrow pH range, specific molecular weight requirement
Emul./solventevaporation	100–200	Homogeneous size	Difficult procedure of washing for nanoparticles isolation
Reverse phase preparation	40	Small size	Nanoparticles isolation
Inverse miniemulsion	150–200	No special gelatin needed	High polydispersity and difficult procedure
Nanoprecipitation	200–350	Simple and straight forward procedure	High amount of surfactant needed

### 3.1 Two-step desolvation

**FIGURE 2:**
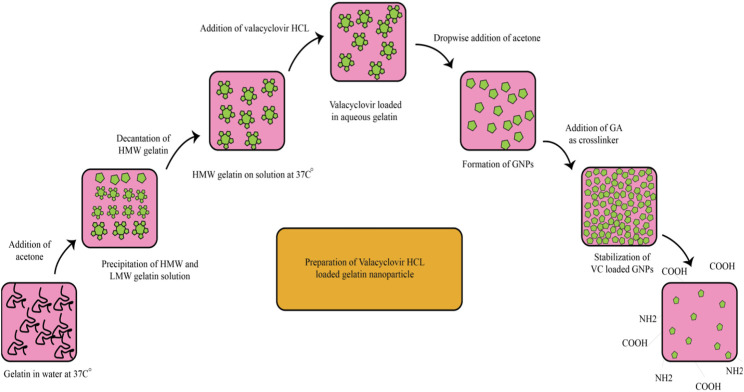
The preparation of valacyclovir (VC)-loaded GNPs via a two-step desolvation process (Sahoo et al., 2015).

Two-step desolvation is a common method used during GNPs development. Desolvation is a thermodynamically driven process for self-assembly of polymeric materials to prepare nanoparticles. Coester et al., in 2008 (Coester et al., 2000) described a method involving the addition of a desolvating agent (acetone) to an aqueous gelatin solution in order to dehydrate the gelatin molecules and produce triple-helix coiled polymeric nanoparticles. In the first desolvation step low molecular weight (LMW < 65 kDa) gelatin components remained in solution, and the HMW precipitate was then redissolved in water. This solution was subjected to a second desolvation step involving careful addition of drops of acetone at monitored pH (2.3–4.0) to avoid the isoelectric point. Small solid nanoparticles with an identical spherical shape were formed by adding a cross-linker and stirring for 12 h. After centrifuging at 10.000 g for 30 min in acetone:water (30:70 ratio), the GNPs were lyophilized 3 times at 2 mbar over the course of 24 h. As shown in Figure 2, the size of the GNPs produced by this method was 110–257 nm (Sahoo et al., 2015).

### 3.2 Simple coacervation

Stable and small-scale particles can be prepared by a simple coacervation method. Coacervation involves the separation of an aqueous solution of a macromolecular polymer into two non-miscible liquid phases, with the lower denser phase containing the macromolecules. To prepare the NPs, salts (sodium chloride or sodium sulfate), or alcohols (ethanol) can be added. Macromolecules with a pronounced charge (like proteins or polyelectrolytes), can undergo complex coacervation. Gelatin molecules are dehydrated at the end and the GNPs are subsequently cross-linked with cross-linking agents like glutaraldehyde (GA) (Yasmin et al., 2017).

### 3.3 Solvent evaporation

This approach involves single or double emulsions, such as oil-in-water (w/o) or double emulsions, water-in-oil-in-water (w/o/w). A high-speed homogenization or ultrasonic mixing technique is used to mix an aqueous phase containing both gelatin and the drug with an oil phase (such as an organic solution of polymethyl methacrylate or paraffin oil), which is then crosslinked with GA or possibly genipin. The solvent can then be evaporated either under reduced pressure or by constant magnetic stirring at room temperature. Then, to remove additives like surfactants, the solidified nanoparticles are aggregated by ultracentrifugation and washed with distilled water. In the final step the material is lyophilized (Sahoo et al., 2015).

### 3.4 Microemulsions

In this technique GNPs were developed using sodium bis (2-ethylhexyl) sulfosuccinate redispersed in n-hexane (AOT) as a surfactant and soaked gelatin solution. Nanoparticles crosslinking by GA and further evaporation of the n-hexane were the final steps for production of GNPs. N-hexane dissolvent of AOT resulted in inverted micelles in which the hydrophobic tails pointed to the outward surface while the hydrophilic head groups are oriented on the inner side surrounding an aqueous core, within which the gelatin and cross-linker are dissolved. As a result, the GNPs are produced inside the inner aqueous core of the inverted micelles due to the cross-linking (Elzoghby, 2013).

### 3.5 Nanoprecipitation

**FIGURE 3:**
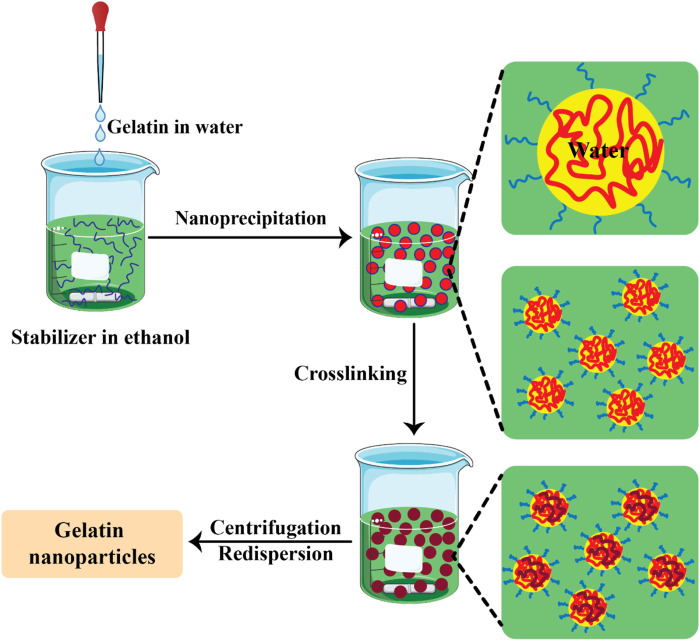
The preparation of gelatin nanoparticles by the nanoprecipitation method (Khan and Schneider, 2013).

In the nanoprecipitation method, an aqueous solution gelatin and the drug is gradually added to ethanol, which contains poloxamer acting as a stabilizer. Afterwards the cross-linker GA is added. Then, a spatially confined distribution occurred due to the miscibility between the solvents. The solvent droplets were disrupted on the nanoscale, and were then stabilized by the stabilizer. When the solvent diffusion was terminated the condensation of the protein took place (Elzoghby, 2013). Figure 3 shows the preparation of GNPs by the nanoprecipitation method.

### 3.6 Microfluidic methods

**FIGURE 4:**
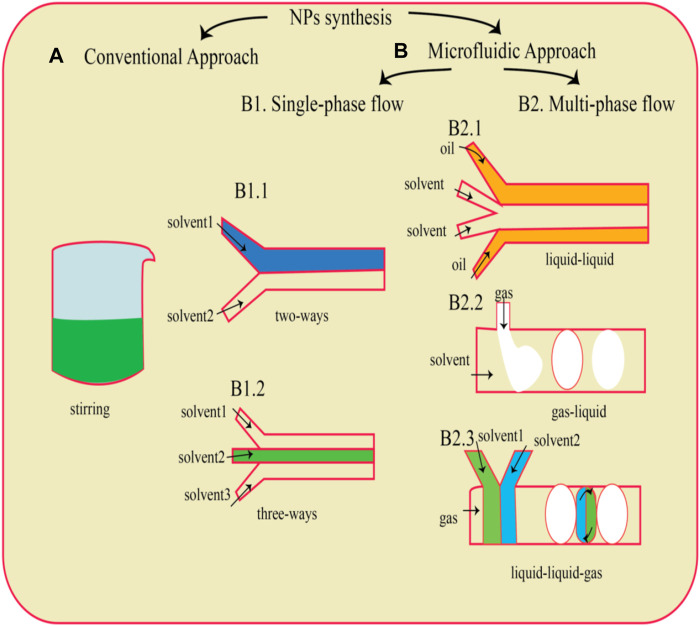
A schematic depiction of a widely utilized traditional technique for nanoparticle (NP) generation namely, the dropwise method **(A)**, is presented. Microfluidic chips **(B)** with diverse designs can be employed for NP production, depending on the type of flow utilized. This includes single-phase flow systems (B1) featuring either two-way (B1.1) or three-way channels (B1.2), as well as multiphase flow configurations (B2) such as liquid–liquid (B2.1), gas–liquid (B2.2), and liquid–liquid-gas (B2.3) systems (Gimondi et al., 2023).

Development of microfluidic approaches and devices are possible due to the collaboration between different fields including physics, chemistry, material science, microelectronics, and fluid dynamics. Increase of products quality along with reduction of cost and time by enhancing different biological and chemical processes are some of the advantages of these devices (Chen and Lv, 2022). These abilities of these devices increase the attention to nanomaterial products. Mixture of metal salt with an agent with reduction ability is a common method for production of metal nanoparticles. The microfluidic method allowed convenient optimization of parameters to obtain a narrower size distribution compared to conventional batch synthesis (Niculescu et al., 2021). Commonly employed microfluidic technologies encompass the T-junction, hydrodynamic flow focusing, staggered herringbone, toroidal mixer, multi-inlet vortex mixers, and others (Xia et al., 2023). The flow behavior within the microfluidic device operates within the laminar regime, where viscous forces predominate. In this regime, laminar flow entails a velocity distribution contingent upon boundary conditions and mass transfer primarily driven by diffusion within the microchannel. Typically, in a hydrodynamic flow-focusing apparatus, a core fluid with a lower flow rate is enveloped by an outer sheath fluid flowing at a higher rate. The heightened flow rate of the outer sheath fluid causes compression within the central flow, thereby diminishing mixing duration. Consequently, this facilitates diffusive mass transfer within the focused stream within microchannels. The reaction occurs predominantly within the centrally focused stream rather than along the channel walls, resulting in a homogeneous distribution of particles. The combination of reproducibility and cost-effectiveness renders microfluidic flow-focusing technology an appealing method for nanoparticle production (Joseph et al., 2022) (Figure 4).

## 4 Self-assembly

Some procedures encourage the self-assembly of gelatin molecules to form nanoparticles.

### 4.1 Chemical modification

The hydrophilic gelatin molecules can be conjugated to various hydrophobic molecules to produce amphiphilic polymers. Hydrophobically modified gelatin can be dissolved in an aqueous solvent, leading to self-assembly into micelle-like nanospheres, where the hydrophobic regions are located in the central part, forming a hydrophobic core which can incorporate hydrophobic drug molecules, along with an external hydrophilic shell.

**FIGURE 5:**
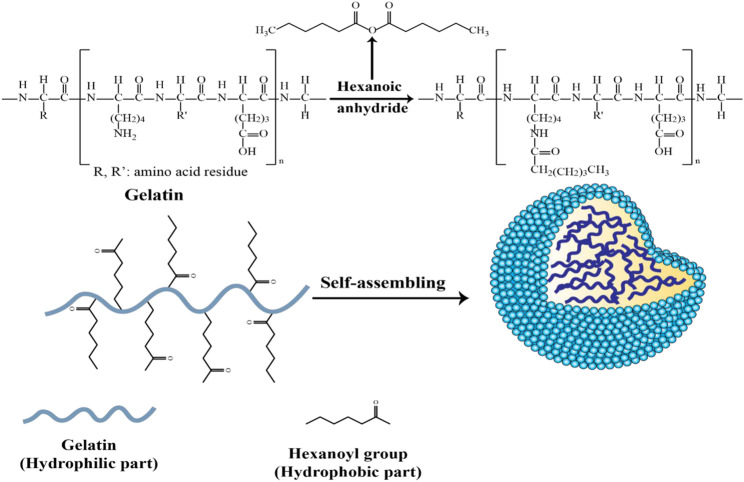
Schematic of the synthesis of self-assembled hexanoyl-modified GNPs (Li et al., 2011).

In another modification of hydrophilic gelatin, hexanoyl anhydride, and alpha-tocopheryl succinate (TOS) were used as hydrophobic groups to be attached to recombinant human gelatin (rHG) (Figure 5) (Li et al., 2011). The nanoparticle core could be loaded with the lipophilic drugs, camptothecin or 17-AAG (17-allylamino-17-demethoxygeldanamycin) by copolymer dilution and sonication. Any free drug was eliminated after centrifugation and dialysis, followed by lyophilization. However, the hexanoyl-modified GNPs demonstrated some instability in aqueous solution, suggesting they may not survive in the bloodstream.

Tanigo et al. (2010) reported that simvastatin could be incorporated into L-lactic acid oligomer (LAo)-grafted gelatin micelles, thus providing water solubility. After gelatin was chemically crosslinked to generate gelatin hydrogels, the micelles were produced. In the presence of collagenase, the hydrogels were enzymatically cleaved to produce water-soluble gelatin leading to the release of simvastatin. In another study using double emulsion or nanoprecipitation methods, the hydrophobic drug, doxorubicin hydrochloride (DOX) was incorporated into amphiphilic gelatin-co-poly (lactide)-1,2-dipalmitoyl-sn-glycero-3-phosphoethanolamine copolymer nanoparticles (Elzoghby, 2013).

### 4.2 Simple mixing

The solutions of gelatin and drugs could be simply blended to produce nanoparticles without the gelatin being chemically modified. Self-assembled GNPs could be formed from mixtures with tea catechins or fractionally purified ellagitannins (PPE) (collectively called tannins) by simple mixing relying on the formation of hydrogen bonds. It has been established that some proteins have tertiary structures with fewer hydrophobic regions that force interactions with the tannin molecules. Gelatin is enriched with proline residues, producing an extensive random coil conformation. Therefore gelatin has regions which can interact with tannin molecules (Elzoghby, 2013).

## 5 Gelatin-based nanoparticles and osteomyelitis

A primary challenge in osteomyelitis therapy is the prolonged presence of the infectious agent within the bone tissue. Inflammation predominantly occurs in avascular regions, leading to spatially diminished bactericidal effectiveness of systemic antibiotic administration and the host immune response (El Zein et al., 2023). The prevailing treatment protocol for both acute and chronic osteomyelitis necessitates a comprehensive approach involving appropriate antimicrobial treatment, surgical intervention, bone reconstruction, and rehabilitation measures (Barakat et al., 2019). Penicillin continues to be the primary antibiotic of choice. For resistant microorganisms, alternatives such as clindamycin, metronidazole, ticarcillin and clavulanic acid, cephalosporins, carbapenems, and vancomycin, often in combination with other antibiotics, may be considered. Empirically, a full course of parenteral antibiotic treatment spanning 4–6 weeks is typically prescribed. Alternatively, initial parenteral therapy may be followed by a 3-week course of oral antibiotics, such as ciprofloxacin and levofloxacin, known for their excellent oral bioavailability and ability to penetrate bone tissue (Bury et al., 2021). In light of this, localized delivery of antibacterial agents emerges as an attractive option for managing chronic osteomyelitis. Historically, antibiotic-loaded acrylic cement (ALAC), polymethylmethacrylate (PMMA) beads, and calcium sulfate pellets have surpassed systemic antibiotic therapy in Europe (Aiken et al., 2024; Jiang et al., 2024; Restrepo et al., 2024). Nevertheless, there are drawbacks associated with the utilization of PMMA and ALAC, notably their nonbiodegradable nature. For instance, during polymerization the PMMA reaction is exothermic, which could result in the destruction of several drugs. Moreover, the antibiotic elution from PMMA could be sub-optimal causing the bacteria to become drug-resistant. Furthermore, patients often undergo secondary surgical procedures for the extraction of the polymeric devices post-antibiotic release, leading to elevated costs and prolonged hospital stays. Failure to remove these devices surgically can result in the formation of foreign bodies, potentially triggering subsequent inflammation and infection (Gibon et al., 2017). Therefore, an ideal device should offer sustained release of local antimicrobial agents. Additionally, it should not hinder the process of new bone ingrowth and ideally promote bone regeneration. Consequently, the integration of resorbable osteoconductive biomaterials with antimicrobial agents appears promising for one-stage surgery in the treatment of osteomyelitis. Therefore, biodegradable scaffolds are preferred for sustained release of drugs to prevent revision surgery. These scaffolds can not only reduce infection, but also boost the regeneration of the bone. Some biodegradable biomaterials, such as bioglass, calcium sulfate, HA-collagen or hydroxyapatite (HAP) are considered to be osteoconductive, and have a chemical composition making them compatible with native bone (Cierny III, 2011; Walter et al., 2012; Lima et al., 2014; Luo et al., 2016; Parent et al., 2016). These materials can be used for the release of antibiotics to reduce infection. For instance, it was shown that the mixture of calcium sulfate beads and antibiotics was useful in osteomyelitis treatment (Papagelopoulos et al., 2006). Nevertheless, the efficiency of these materials is limited by the volume of debrided bone (above a critical size) that needs to be regenerated in osteomyelitis. Regarding infected bone defects, scaffold biodegradation speed should be in line with the time needed for the removal of the bacteria, and then to allow tissue regeneration (typically 1–4 months). Furthermore, neovascularization should be promoted by the scaffold. Neovascularization required several sub-processes including osteoblast movement and stem cell boosting at the site of the bone defect. Studies also showed that adding elements such as silicon or boron in to these bioceramic materials have increased the bone regeneration rate. The antibiotic teicoplanin was released over 20–30 days from particles of borate-containing bioactive glass prepared from ammonium phosphate and chitosan, and this preparation resulted in increased bone regeneration in a *in vivo* model of osteomyelitis over 12 weeks of implantation (Zhang et al., 2010).

Controlled infection offered one-step structural support for the ingrowth of bone tissue (Krishnan et al., 2020). After 3 months, the stimulant was rapidly absorbed, and there was still a vacancy in the bone at the defect site. Therefore, the vancomycin-containing nanocomposite fibrous scaffold acted as a bi-functional graft, both decreasing bacterial infection, and regenerating the bone in osteomyelitis.

**FIGURE 6:**
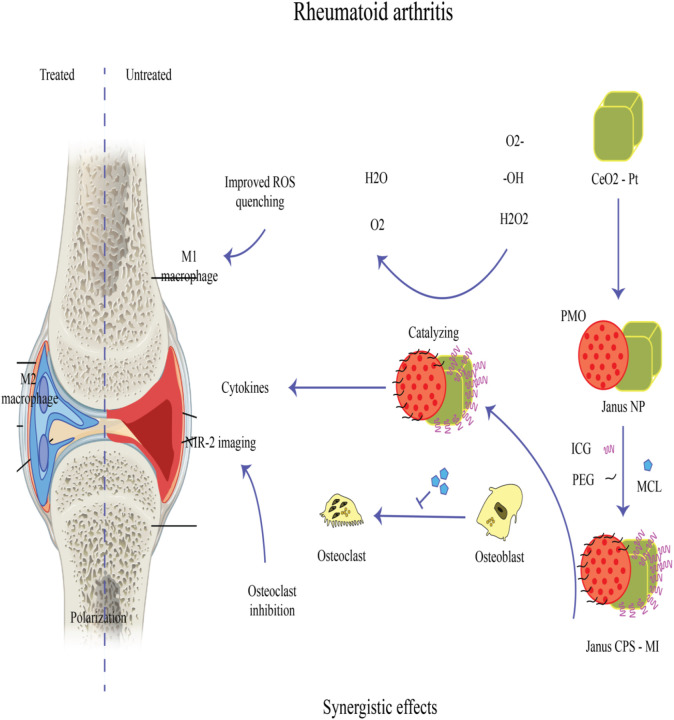
A Janus nanoplatform (Janus-CPS) has been developed for the simultaneous early detection and combined treatment of rheumatoid arthritis (RA). This platform consists of CeO2-Pt nanozyme on one side and periodic mesoporous organosilica (PMO) on the other. Micheliolide (MCL), known for its anti-osteoclastogenesis properties, is encapsulated within the mesopores of PMO to synergistically complement the soothing properties of nanozymes, thereby effectively managing RA. To achieve the desired efficacy in early RA detection, Janus-CPS loaded with indocyanine green (ICG) utilizes NIR-II fluorescence imaging (Huang et al., 2024).

There have been studies on strontium-incorporated hydroxyapatite (Sr-HAP) in orthopedics, dentistry, and bone tissue engineering. HAP has a Ca:P ratio of 1.67, and can act as a useful replacement for the apatite naturally found in bones. Different studies have been conducted on HAP combined with organic or inorganic polymer systems, as well as for drug delivery methods including hydro-gels, scaffolds, coatings, thin films, etc. The extensive adaptability of HAP and its compatibility with many systems, allows it to act as an appropriate niche for bone mineralization. Additionally, HAP have favorable features including its bio-degradability, and -compatibility along with osteoconductive nature, supporting cell attachment via adhesion and proliferation (Kumar R. et al., 2011; Olad and Azhar, 2014). Chitosan (deacetylated chitin) and gelatin (the denatured form of collagen) are widely used in bone reengineering as these proteins have high similarity with bone collagens (Olad and Azhar, 2014). Moreover, when gelatin and HAP are mixed at the ratio of 65:35 they imitate the natural bone composition. In addition to the eco-friendly and non-toxic properties of chitosan and gelatin, no immunologic reactions are stimulated (Jiang et al., 2012; Yao et al., 2015). In comparison to synthetic polymers, natural polymers degrade rapidly leading to loss of their mechanical properties. Porous co-polymer systems composed of chitosan and gelatin combined with HAP have been used in tissue repair as well as drug delivery systems. Extensive research has been conducted on their ability to deliver antibacterial and anti-inflammatory drugs, carrier systems for gene delivery, molecular mechanics, anti-cancer agents, etc. (Bose and Tarafder, 2012; Zhang et al., 2012; Kalil et al., 2014). Their high surface energy and bioinertness have led them to be used as coatings for titanium metallic implants (Oldani and Dominguez, 2012). Due to their inert behavior, titanium implants are renowned for having a prolonged lifespan of at least 15 years with no negative or hazardous reactions. However due to the surface inconsistency, a fibrous capsule will be produced surrounding the metal, which protects infections from attack by the host immune response. As a result, the delivery of antibacterial drugs is difficult, which prevents the bone from being regenerated (Dawes et al., 2010). Sun et al. (2023) illustrated that manganese-doped albumin-gelatin composite nanogels loaded with berberine exhibited the capability to target inflammatory joints. This targeting ability stems from albumin’s inherent high affinity for secreted protein acidic and rich in cysteine (SPARC), which is notably overexpressed at the inflammatory sites of gouty arthritis. Both *in vitro* and *in vivo* experimental findings demonstrated that this composite formulation yielded superior therapeutic outcomes, effectively alleviating oxidative stress and dampening inflammation (Sun et al., 2023). Another study documented the efficacy of injectable vancomycin (Van)-loaded gelatin/nanohydroxyapatite (Gel/n-HA) composite microspheres (VM) in modulating inflammation in a rabbit model of osteomyelitis. Injectable VM demonstrated a successful treatment outcome by virtue of its targeted antibacterial action, inflammation modulatory effects, recruitment of osteoblasts, and promotion of bone regeneration, thus effectively addressing osteomyelitis (Zhang et al., 2023). In a recent study, a novel hyaluronic acid/gelatin nanocomposite hydrogel coating was applied to titanium-based implants in a rat model of implant-associated infection. This coating demonstrated the ability to remove biofilms and regulate oxidative stress and inflammatory responses, thereby promoting osseointegration (Ding et al., 2023). Figure 6 provides an overview of the mechanisms of nanoparticle (NP) treatment for rheumatoid arthritis, highlighting its role in addressing the inflammatory aspects of the disease.

Encouraging potential in the management of patients with persistent osteomyelitis infection a possible solution for non-union fractures to stimulate bone regeneration (David and Nallaiyan, 2018). David et al. prepared vancomycin-loaded chitosan–gelatin polyelectrolyte complex combined with gelatin-SrHAP to produce HV scaffolds at different concentrations HV1-0.5% and HV2-1%. Scaffolds of HG, HV1, and HV2 were efficiently formed on Cp-Ti by attachment using dopamine linkers, which formed bidentate coordination by NH bonds. In addition, the HV2 scaffold greatly outperformed the Cp-Ti, HG, and HV1 scaffolds in terms of cell viability. Vancomycin did not have any harmful effect on the cells; on the contrary, it increased cell proliferation and spreading.

Ciprofloxacin (CFX) hydrochloride (1-cyclopropyl-6-fluoro-4-oxo-7-piperazin-1-yl-1,4-dihydroquinoline-3-carboxylic acid hydrochloride) is from second-generation fluoroquinolones and is a wide spectrum antibiotic influence on both gram negative and positive bacteria. It has received approval to treat respiratory tract infections, different bone and joint infections, urinary tract infections and some topical infections (Tjan-Heijnen et al., 2001). Pandey et al. (2016) designed a system composed of gelatin-based chemically cross-linked cryogel, CaCO3 microspheres, and CFX for treatment of osteomyelitis and osteoporosis. The porosity, pore volume, swelling ratio, swelling kinetics, compression strength, and *in vitro* degradation rate of the produced cryogel, were all correlated with the amount of gelatin present, the time of the freezing process, and the number of freeze-thaw cycles. After an initial burst release of CFX, sustained release continued over 21 days, and the concentration was maintained higher than the MIC for the entire research phase. The *in vitro* antibacterial activity measurement against *S. aureus* and *Escherichia coli* showed the following zones of inhibition on Mueller-Hinton agar plates on days 1, 3, 5, and 7: 33, 30, 28, and 27 mm for *S aureus*; 43, 37, 37, and 36 mm for *E. coli*, respectively. As opposed to a 2D surface, the cell viability, the number of cells within the growth phase, and the alkaline phosphatase activity in rat osteoblasts cultured in the cryogel were considerably higher. They concluded that this microsphere incorporated, CFX-loaded, industrially amenable cryogel system could be beneficial in osteomyelitis and osteoporosis treatment.

A number of organic and inorganic particles, including silica nanoparticles, nano-hydroxyapatite, and poly (lactic-co-glycolic acid) (PLGA) microparticles, have been investigated as the drug carriers (Gentile et al., 2016; Qiu et al., 2016). Mesoporous silica nanoparticles (MSNs) have been explored in tissue engineering as potential nano-additives. This is because they provide many advantages, including adjustable particle and pore sizes, a considerable drug loading capacity, and superb biocompatibility (Zhou et al., 2015; Chen et al., 2016).

Zhou X. et al. (2018) developed Van@MSNs which was composed of both vancomycin and MSNs, in combination with a gelatin matrix to create a composite scaffold. The gelatin-based composite scaffolds were found to have a very porous microscopic structure. Improvement of compression property of composite structures occurred using MSNs. Additionally, *in vitro* studies revealed that the Van was released from Van@MSN-incorporated composite scaffolds continuously almost without an initial burst. This efficiently prevented *S. aureus* growth bone mesenchymal stem cells (BMSCs) homeostasis functions along with their differentiation and promotion were not adversely affected by the drug-loaded composite scaffold, indicating acceptable biocompatibility. *In vivo* study indicated increased bone regeneration along with reduction of bacterial contamination following utilizing of these antibiotic-loaded agents. The synthetic Van@MSNs/Gelatin composite scaffold could be a suitable biomaterial for treating infected bone by providing a localized and sustained-release of antibiotics. Another study demonstrated that tannic acid–mineral nanoparticles embedded within a gelatin-based cryogel notably improved both the quality and quantity of newly formed bone (Song et al., 2024).

**FIGURE 7:**
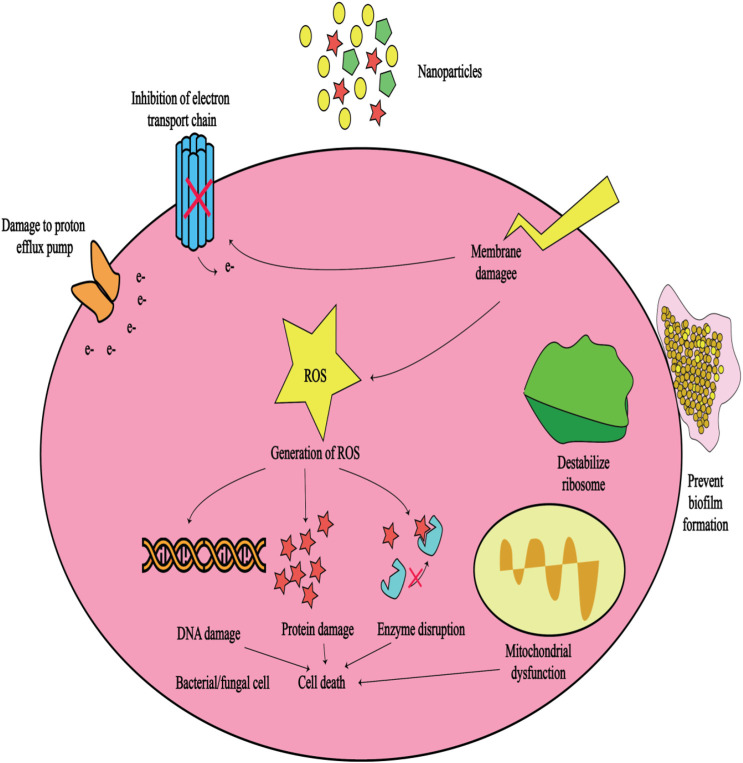
Schematic of the potential mechanisms of antibacterial drugs delivered by gelatin nanoparticles that can be useful in the treatment of infectious diseases (Madkhali, 2023).

For the controlled release of vancomycin, Zhou et al. created gelatin scaffolds with varying concentrations of β-TCP (0%, 10%, 30%, and 50%). These scaffolds were denoted G-TCP0, G-TCP1, G-TCP3, and G-TCP5, respectively (Jiang et al., 2012). The Van release profile was examined using the Kirby-Bauer method. They then tested them in rabbit models of chronic osteomyelitis. The infected bone defects were implanted with scaffolds following complete debridement. The effectiveness of infection reduction and the repair of bone defects were investigated using radiographs and histological analyses. The gelatin/β-TCP scaffolds revealed a homogeneously interconnected 3D porous structure. The G-TCP0 scaffold had the longest Van release period of 8 weeks. The Van release time of the composite scaffolds containing more β-TCP was shorter as its content increased. Within 3 weeks, Van was fully released from the G-TCP5 scaffold. The G-TCP3 scaffold was found to be the most effective in reducing infection and healing bone defects in rabbits with osteomyelitis. The G-TCP3 scaffold performed well in terms of porosity, connectivity, and controlled release. As a result, this scaffold could be employed to treat chronic osteomyelitis lesions. Table 2 lists some different gelatin composites that have been investigated in the treatment of osteomyelitis. Furthermore, Figure 7 illustrates the potential mechanisms of antibacterial drugs delivered via gelatin nanoparticles, which can be beneficial in treating various infectious diseases. These mechanisms encompass four aspects: antibiotic delivery, targeting bacterial toxins, impairing bacterial cell walls and membranes, and disrupting bacterial DNA, proteins, and enzymes (Huang et al., 2024).

**TABLE 2:** Different gelatin composites used in the treatment of osteomyelitis.

Gelatin composite	Target microorganism	Model (in vitro, in vivo)	Mechanism of activity	Results	Ref.
Gelatin-based scaffolds loaded with silk fibroin nanoparticles and β -tricalcium phosphate	—	*In vitro*	- Controlled release in drug delivery systems - Osteoblast differentiation	- Accelerate osteoblast differentiation and increase the healing rate of bone tissues	Llorente et al. (2024)
Gelatin-based hydrogel incorporating mesoporous silica nanoparticles (MPS-NPs) loaded with rifampicin (RIF) and levofloxacin (LEV)	*Mycobacterium bovis*	*In vitro* *In vivo*	- Sequential release of drugs	- Minimum inhibitory concentrations value against *M. bovis* for LEV-loaded and RIF-loaded MPS-NPs were 6.50 and 1.33 µm/mL, respectively - WST-1 test confirmed the biocompatibility and safety of the developed vertebral hydrogel bioimplant - Histological and immunohistochemistry micrographs showed the progress in healing process with the bioimplant - Besides, loading of LEV and RIF in the implants declined the presence of the giant macrophages clusters as compared to control groups	Luo et al. (2024)
Ciprofloxacin-loaded gelatin fibers	Spectinomycin-resistant *E. coli*	*In vitro*	- Hydrophobic cargo release	- An inhibitory effect on bacterial growth in a solid medium was observed	Song et al. (2024)
Nanofiber gelatin scaffolds containing curcumin/vancomycin	*S. aureus* (MRSA)	*In vitro*	- Slow drug release pattern of gelatin scaffolds	- Ability to treat bone infections caused by MRSA. - Slow release of vancomycin from gelatin provided a long antibacterial effect for 21 days - Gelatin scaffolds had favorable biocompatibility and significantly promoted the Mesenchymal stem cells adhesion and proliferation	Yekani et al. (2023)
Gelatin composite gel particles loaded with zinc oxide and silver nanoparticles	*S. aureus*	*In vitro* *In vivo*	- The targeted release of payloads facilitated by enzymatic degradation triggered by gelatinases in *S. aureus* and the well-distributed NPs in the gel network - The silver and zinc oxide NPs as well as the released ions could cause the disruption of cell membranes, bind with the deoxyribonucleic acid molecules, and release reactive oxygen species inside the cell, resulting in the bacterial cell lysis and the deoxyribonucleic acid fragmentation	- The selective antibacterial activities of composite gel particles on *S. aureus* via enzymatic degradation and highlights the importance of preparing antimicrobial agents in gelatin networks to proceed targeted release against bacteria	Hui-Zhong et al. (2023)
Vancomycin-loaded gelatin/nanohydroxyapatite (Gel/n-HA) composite microspheres (VM)	*S. aureus*	*In vitro* *In vivo*	- The bacterial cell wall demonstrated severe destruction, causing cytoplasmic outflow - Leakage of the cytoplasmic substrate from the broken cell envelope	- Bone tissue regeneration was observed in the treated groups with increased healing time - Injectable VM exhibited a successful treatment outcome via targeted antibacterial, inflammation modulatory, osteoblast recruitment, and bone regenerative properties to treat osteomyelitis	Zhang et al. (2023)
Alginate-di-Aldehyde-Gelatin Gels (ADA-GEL) loaded with clindamycin	*S. aureus*	*In vitro*	- Continuous release with a reduced burst release	- Dual release of clindamycin from ADA-GEL beads was shown to be possible over an extended period of time (up to 4 weeks) with antimicrobial effective concentrations (CLI 25-fold above the MIC) - The tissue compatibility of ADA-GEL was demonstrated using various biocompatibility tests in cell culture with MG-63 cells	Schrade et al. (2022)
Gentamicin-loaded magnetic gelatin nanoparticles (GMGNPs)	—	*In vivo*	- GMGNPs have controlled drug release profile	- Based on *in vivo* and *ex vivo* studies, after six doses of GMGNPs treatment, abscess began to heal and the integrity of periost and bone began to reconstruct - GMGNPs could provide efficient therapy for osteomyelitis	Ak et al. (2021)
Nano-hydroxyapatite gelatin scaffold reinforced with poly-L-lactic acid yarns coated with silica- vancomycin	MRSA (ATCC 43300)	*In vitro* *In vivo*	- Peptidoglycan production for the synthesis of the bacterial cell wall was inhibited by vancomycin	- Controlled infection - Offered one-step structural support for the ingrowth of bone tissue	Krishnan et al. (2020)
Gelatin-agarose scaffold with the addition of glass nanoparticles containing ciprofloxacin	—	*In vitro*	- Incorporation of glass nanoparticles in the scaffolds improved their drug release profiles and rates	- This scaffold acted as a potent treatment for osteomyelitis	Ali et al. (2020)
Wet spun poly (ε-caprolactone) fibers and films plus vancomycin-loaded gelatin microspheres	*S. aureus* (ATCC 29213) S. epidermidis (ATCC 35984)	*In vitro*	- Van hydroxyl groups and gelatin amino groups formed a hydrogen bond	- High antibacterial activity against *S. aureus* and S. epidermidis	Aksoy et al. (2019)
Porous silica nanoparticle/gelatin composite scaffold loaded with vancomycin	*S. aureus*	*In vitro* *In vivo*	- Incorporated efficient antibacterial components Van was released from the scaffold to prevent bacterial growth	- An effective biomaterial for treating bone infection. Van@MSN/gelatin composite scaffold allowed localized and sustained antibiotic release and further enhancement of bone structure improvement	Zhou et al. (2018a)
Titanium-fabricated chitosan/gelatin-SrHAP scaffold	MRSA) (ATCC 43300) MSSA (ATCC SA113)	*In vitro*	- Local drug release at the infection site	- Encouraging potential in the management of patients with persistent osteomyelitis infection - A possible solution for non-union fractures to stimulate bone regeneration	David and Nallaiyan (2018)
Vancomycin-gelatin	*S. aureus* RN4220	*In vivo*	- Reduction of bacteremia due to the both intra and extracellular release of vancomycin in larvae	- Localized delivery system to the involved area	Zhang et al. (2018)
Gelatin/β-tricalcium phosphate composite scaffold containing vancomycin	MRSA	*In vitro* *In vivo*	- Treated infected bone defects - Sustained delivery of vancomycin by diffusion after gelatin matrix degradation	- Positive results in controlling infection - Repair of bone fracture in a model of persistent MRSA osteomyelitis - Could be used to clinically treat osteomyelitis	Zhou et al. (2018b)
Vancomycin incorporated chitosan/gelatin coatings on a TiO2–Sr-HAP surface	MRSA MSSA	*In vitro*	- Prevented adhesion of bacteria and improved cellular communication at the bio-interface - Combined effect of chitosan and nanostructured coating	- A potentially effective method for controlling and eliminating osteomyelitis at the infection site while promoting bone mineralization	Nancy and Rajendran (2018)
Hydroxyapatite-gelatin-silica composite	*S. aureus* *S. enteritidis* *P. aeruginosa* *E. coli* *Serratia liquefaciens*	*In vitro*	- Prevented peptidoglycan production in the bacterial cell wall - The interaction between HAP nanoparticles and bacteria was reduced when the nanoparticles were covered with gelatin-silica	- Promising as bone grafts for osteomyelitis - Toxic to bacteria	Uskoković et al. (2017)
A chemically cross-linked cryogel system based on gelatin loaded with CaCO3 microspheres and ciprofloxacin hydrochloride	*S. aureus* *E. coli*	*In vitro*	- Burst release of CFX embedded at the cryogel matrix edges - Controlled release from matrix interior	- Possible therapy for osteoporosis and associated osteomyelitis	Pandey et al. (2016)
Ciprofloxacin-loaded gelatin-hydroxyapatite scaffolds	MRSA MSSA	*In vitro*	- Ciprofloxacin could cross human cell membranes and eliminate intracellular bacteria	- The gelatin-HAP scaffolds loaded with ciprofloxacin were cytocompatible and could target both intracellular or extracellular *S. aureus* - Good potential as a local drug delivery system	Krishnan et al. (2015)
Bovine hydroxyapatite- gelatin-glutaraldehyde-gentamicin composite (BHA-GEL-GA-GEN)	*S. aureus* (ATCC 25293)	*In vitro* *In vivo*	- Gentamicin had anti-inflammatory effect by inhibiting NADPH oxidase activity in neutrophils	- Released high concentration of GEN over 28 days - In osteomyelitis therapy, BHA and GEL functioned as bone remodeling templates	Budiatin et al. (2014)
β-tricalcium phosphate (TCP) scaffold doped with gentamicin and combined with a gelatin/genipin hydrogel (G-TCP)	*S. aureus* (ATCC 25923)	*In vitro* *In vivo*	- Bacterial adhesion and proliferation could be decreased by gelatin/genipin - The absence of edema, decreased osteolytic changes, and the growth of new cortical bone at the site of infection, all indicated that G-TCP drastically reduced bone infection	- The G-TCP system had unexpected benefit in the treatment of osteomyelitis and should be further investigated in clinical settings	Wu et al. (2013)
Gelatin/β-TCP composite plus vancomycin	*S. epidermidis* RP62A	*In vitro* *In vivo*	- Promoted bone regeneration Acted as a controlled release carrier for bone morphogenetic protein 2 (BMP-2)	- A promising candidate for controlled vancomycin release in persistent osteomyelitis. Local therapeutic drug release over a long period of time	Zhou et al. (2012b)

Although nanoparticles offer innovative approaches to combat bacterial infections and may potentially mitigate antibiotic resistance, it is crucial to acknowledge that resistance mechanisms can still emerge. For example, bacteria may evolve mechanisms to hinder the attachment of nanoparticles or to efflux them (Hetta et al., 2023). Hence, GNPs leveraging their aforementioned characteristics, can serve as carriers to encapsulate metal nanoparticles like silver or gold nanoparticles, renowned for their antimicrobial efficacy. The resultant hybrid nanoparticles offer heightened antibacterial activity and have the potential to diminish antibiotic resistance by offering an alternative to conventional antibiotic therapies (Khan and Rasool, 2023). Finally, GNPs have been widely employed for delivering diverse antibiotics. They serve the dual purpose of safeguarding these drugs from degradation in the physiological milieu and facilitating targeted, controlled release, thereby potentially augmenting their efficacy while minimizing side effects (Figure 7). Numerous studies (Table 2) have successfully loaded antibiotics such as vancomycin, rifampicin, levofloxacin, ciprofloxacin, and gentamicin into GNPs for targeted delivery to sites of osteomyelitis infection, resulting in enhanced antibacterial activity.

## 6 Conclusion

Gelatin is an optimal delivery system for sustained release of different biomolecules used widely in regenerative medicine researches. This macromolecule is a suitable system for drug delivery due to its low cast, availability, biodegradability, and biocompatibility. However, induction immune response due to their potential antigenicity and the risk of contamination of gelatin with different pathogens especially in animal-origin forms along with their low batch-to-batch reproducibility have challenged the use of these molecules in medicine. Development of GNPs increases the hopes for using gelatin in medicine with more efficacy and less side effects as these unfavorable effects have not been seen in studies yet. But safety of starting material and cross-linking agents should be studied more in further researches. Moreover, the efficacy of these particles could be improved significantly by conjugation of other materials to cover these particles limitations. Despite these promising findings, using these agents have not become a routine in clinic yet. Due to the favorable release profile of these agents, delivery of antibiotics such as vancomycin to the infection site via this module have increased the succession rate of osteomyelitis eradication (Zhou et al., 2012a). Considering that gelatin can be chemically modified, and combined with different growth factors and several antibiotics, gelatin-derived composites could be promising candidates for bone tissue engineering and therapeutics. With the recent development of 3D printing technology, substantial improvements in the biomaterial field have already been made. To date, a number of *in vivo* research studies have been conducted to evaluate the utility of gelatin-based composites in the management of osteomyelitis. Modification of GNPs surface via different methods such as coating with ligands and polyethyl glycols along with using amine derivatives on the surface of these particles are used for recruitment of these particles in different fields of medicine. GNPs also have better stability in biological fluids which provide better controlled drug release in the targeted sites compared to other colloidal carriers. Gelatin-based composites were found to have satisfactory performance in the control of infection, as well as the promotion of bone defect repair in chronic osteomyelitis models. Therefore, this review of the available evidence provides a new insight into gelatin-derived composites with controlled release of antibiotics. When combined with the stimulated repair of bone defects, these may be a sign of the future development of osteomyelitis treatment.
